# In vitro and in silico study of the endosulfan degradation by *Bacillus subtilis* sp. strain UAMC

**DOI:** 10.1007/s10532-026-10312-y

**Published:** 2026-05-19

**Authors:** Adriana Casanova, Sergio Hernández, Diego A. Esquivel-Hernández, Sergio Revah, Irmene Ortíz

**Affiliations:** 1 Posgrado en Ciencias Naturales e Ingeniería, Universidad Autónoma Metropolitana-Cuajimalpa, Av. Vasco de Quiroga 4871, Col. Santa Fe, C.P. 05348 Mexico City, México; 2 Depto. Procesos y Tecnología, Universidad Autónoma Metropolitana-Cuajimalpa, Av. Vasco de Quiroga 4871, Col. Santa Fe, C.P. 05348 Mexico City, México

**Keywords:** Endosulfan, *Bacillus subtilis*, Biodegradation, Molecular docking, Bioremediation

## Abstract

The widespread use of endosulfan, a persistent organochlorine pesticide, has raised significant environmental and health concerns due to its toxicity and potential for bioaccumulation. This study investigated the degradation pathway of endosulfan by *Bacillus subtilis* sp. strain UAMC, combining in vitro and in silico approaches. These degradation assays revealed preferential degradation of α-endosulfan (97.6%) over β-endosulfan (69.45%), and identified key metabolites. The toxic endosulfan sulfate metabolite was not detected, suggesting a hydrolytic degradation mechanism. Molecular docking simulations highlighted interactions between endosulfan metabolites and enzymes such as laccase (3ZDW), which possess the necessary structural plasticity to recognize and transform endosulfan, even though they were not evolved for that specific purpose. These findings provided a mechanistic understanding of endosulfan biodegradation and underscore the potential of *Bacillus subtilis* sp. strain UAMC for pesticide bioremediation applications.

**Supplementary Information:**

The online version contains supplementary material available at 10.1007/s10532-026-10312-y.

## Introduction

The use of pesticides has played a critical role in driving significant increases in agricultural productivity and ensuring global food security. However, their widespread application has led to substantial environmental contamination, adversely affecting ecosystems and human health (El-Sheikh et al. 2022; Sathishkumar et al. 2021). These chemicals permeate ecological compartments, including soil, surface water, groundwater, and the atmosphere, triggering bioaccumulation and biomagnification processes that threaten biodiversity and disrupt ecosystem function (Tang et al. 2025; Zhou et al. 2024).

According to official reports, in 2022, total pesticide usage worldwide reached 3.7 million tons (FAOSTAT 2024). This corresponded to an average application of 2.4 kg per hectare of cropland, underscoring the prevalence of intensive agricultural practices (FAOSTAT 2024). Notably, organochlorine pesticides accounted for approximately 40% of total use (da Silva Júnior et al. 2024). In particular, endosulfan is a cyclodiene-class insecticide widely used due to its high efficacy in pest control (e.g., *Hypothenemus hampei*, *Nezara viridula*, and *caterpillars*) (Casanova et al. 2021). Commercial endosulfan consists of a mixture of two stereoisomers, α-endosulfan and β-endosulfan, in a 7:3 ratio (Casanova et al. 2021; Vandenberg et al. 2020). Once released into the environment, endosulfan undergoes hydrolysis, photodegradation, and biodegradation, contributing to its breakdown and eventual mineralization (i.e., conversion to CO_2_ and water). However, these pathways also generate metabolites such as endosulfan sulfate, endosulfan lactone, and endosulfan ether (Kucukcongar et al. 2024; Mudhoo et al. 2019; Singh et al. 2017). Although these metabolites keep the chlorinated structure of the parent compound, studies indicate they are generally less toxic than the original endosulfan molecule (Sathishkumar et al. 2021; Singh et al. 2017). Endosulfan, along with its isomers and degradation products, has been identified as a persistent organic pollutant (POP). This classification led to their addition to the global elimination list under the Stockholm Convention in 2011, which emphasizes these harmful substances for the protection of human health and ecosystems (Shunthirasingham et al. 2024).

Understanding endosulfan degradation pathways and contributing factors is crucial for evaluating its long-term environmental impact. As with other pesticides, biodegradation appears to involve a series of enzymatic reactions, including oxidases, hydrolases, and oxidoreductases (Chaudhary et al. 2024; Mandpe et al. 2022). This information is vital for developing more effective remediation strategies and avoiding their persistence in the environment (Mitra et al. 2024; Takeshita et al. 2023; Webster et al. 2024).

Since enzymes act on metabolites through specific substrate interactions, studying these interactions at the molecular level is key for improving biodegradation processes (Sarker et al. 2021). Bioinformatics and in silico analysis have emerged as powerful tools, offering innovative approaches that complement traditional experimental studies (Mitra et al. 2024). By providing a predictive framework, these tools may help identify enzymes with a higher affinity and specificity for endosulfan degradation metabolites, which is essential for developing effective bioremediation strategies. In recent years, some studies involving microbial strains have been identified for their capability to degrade endosulfan, including some species from the genera *Pseudomonas*, *Rhodococcus*, *Stenotrophomonas*, *Alcaligenes*, and *Flavobacterium* (Chauhan et al. 2016; Kafilzadeh et al. 2015; Kong et al. 2013; Sakthivel et al. 2022; Zaffar et al. 2018). The bioremediation potential of these microorganisms is based on their ability to produce specific enzymes that break down the endosulfan complex structure and its toxic sulfite group (Singh et al. 2019). Previous studies have identified enzymes such as hydrolases, monooxygenases, and oxidoreductases, including laccases, that play important roles in the initial hydrolysis and subsequent oxidation steps of the degradation pathway (Chia et al. 2024; Ortiz-Hernandez et al. 2013).

Even with these findings, the detailed enzymatic mechanisms and the step-by-step formation of endosulfan intermediate metabolites, particularly in highly adaptable organisms such as *Bacillus subtilis,* are still unclear. This highlights the importance of combining both experimental (in vitro) and computational (in silico) approaches to gain a more complete picture of the process (Mudhoo et al. 2019).

Building on this foundation, this study aims to identify potential enzymes and their metabolites involved in endosulfan degradation through a combined in silico and experimental approach.

## Materials and methods

### Inoculum preparation

*Bacillus subtilis* sp. strain UAMC (*B. subtilis* sp. UAMC) was isolated from the “Cinturón hortícola platense” in La Plata, Argentina (34° 51′ 23.897″ S, 58° 16′ 19.198″ O) and previously reported (Casanova et al. 2024). The strain was cultured in a 500 mL Erlenmeyer flask containing 200 mL of Luria–Bertani medium and 2 mL of the strain preserved in glycerol (Casanova et al. 2024). An initial concentration of 5 mg/L of endosulfan was added, and the culture was incubated for 72 h at 150 rpm and 30 °C. Then the endosulfan concentration was increased every 72 h until a concentration of 20 mg/L was reached. Once the strain was acclimated, the inoculum was prepared by centrifuging it at 10,000 rpm for 10 min. The pellet was then resuspended in 20 mL of sterile distilled water. This washing procedure was repeated three times under the same conditions to remove any residual medium and endosulfan from the cells. The final solution served as the inoculum for the degradation assays detailed below.

### Degradation assays

The degradation tests were performed in duplicate using 125 mL flasks, each contained a total working volume of 30 mL, comprising 27 mL of mineral medium (in g/L): 5.97 Na_2_HPO_4_, 0.01 CaCl_2_·H_2_O, 2.27 KH_2_PO_4_, 0.99 (NH_4_)_2_SO_4_, 0.025 FeSO_4_ ·7H_2_O, and 0.5 MgSO_4_·7H_2_O, and 3 mL (10% v/v) of high-density bacterial inoculum. This inoculum consisted of a mature culture of *B. subtilis* sp. UAMC grown to the late exponential phase to ensure high initial metabolic activity. Endosulfan (Sigma-Aldrich, isomers α and β, ratio 7:3, 99.9% purity) was added to a final concentration of 20 mg/L. The culture was incubated under constant agitation at 150 rpm and 30 °C for approximately 33 days. The flasks were sealed with a Mininert® valve that allowed periodic sampling of gas from the headspace, as described below (Casanova et al. 2021). To ensure the reliability of the results, two types of negative controls were included in the experimental design. The first was a biotic endogenous control that contained no external carbon source in the mineral medium inoculated with the bacteria, but did not contain endosulfan or any other external carbon or energy source. This control allowed the determination of basal cellular respiration. The second was an abiotic control, where no microbes were added to the flask. However, 20 mg/L of endosulfan was present in the absence of bacterial inoculation, allowing the evaluation of the abiotic (chemical or physical) endosulfan degradation under the experimental conditions.

The quantified parameters, including CO_2_, biomass, residual endosulfan concentration, and metabolite identification, are outlined in the next section. CO_2_ production was monitored periodically, and destructive analyses of residual endosulfan and biomass were conducted on duplicate flasks at set time intervals. This method allowed for detailed tracking of how endosulfan was converted into various metabolites throughout the experiment.

### In vivo analytical methods

#### Carbon dioxide quantification.

The conversion to CO_2_ served as an indirect indicator of the metabolic activity of *B. subtilis* sp. UAMC. Headspace samples (100 μL) were collected three times a week (at 48 to 72 h intervals) and were analyzed by gas chromatography using a GOW-MAC Series 550 system, USA, with a thermal conductivity detector (GC-TCD) and an Altech Concentric CTR1 column, using helium as the carrier gas, as described previously (Casanova et al. 2021).

#### Biomass quantification.

Biomass was quantified using the dry weight method as described by Casanova et al. (2024). Flask contents were filtered through nylon membranes (0.45 mm), washed with dichloromethane, and then dried to constant weight.

### Quantification of residual endosulfan and metabolite identification

#### Liquid–liquid extraction

After filtration, the liquid phase was extracted with 33 mL of dichloromethane using magnetic stirring for 10 min. The organic phase was separated, filtered, and concentrated to 10 mL using hexane through rotary evaporation at 35 °C and 50 °C, following the procedure described previously (Hernández-Ramos et al. 2019).

#### Residual endosulfan quantification and metabolite identification

Concentrated extracts were analyzed using the EPA 8270D method by gas chromatography-mass spectrometry (Agilent 6890 N, MSD 5975B, USA) equipped with an SGE 5 MS capillary column, following the procedure described by Casanova et al. (2021). Briefly, the oven’s initial temperature was 90 °C, then gradually increased to 250 °C at a rate of 5 °C min ^−1^. Helium was used as the carrier gas throughout the analysis. The detector and injector temperatures were maintained at 220 °C and 250 °C, respectively, to ensure consistent performance during the runs.

For metabolite detection, data were acquired in scan mode over a mass to charge (m/z) range of 50–450 z/m, using an ionization energy of 70 eV. Identification of the detected compounds was performed by comparing their spectra with those in the NIST05 Mass Spectral Library.

### In silico methods

#### Genome sequencing

*B. subtilis* sp. UAMC genome was previously sequenced and reported by Casanova et al. (2024). Briefly, the pure strain was cultivated under standard conditions, and genomic DNA was extracted following quality control verification. Whole genome sequencing was performed using an Illumina MiSeq v2 system (Micro run, 300 cycles).

Also, raw reads were processed and assembled using the KBase bioinformatic platform, following the workflow described in the published genome announcement (Arkin et al. 2018; Casanova et al. 2024). The draft genome annotation identified sequences potentially related to xenobiotic degradation, oxidative stress, and hydrolytic enzyme activity (Casanova et al. 2024). This annotated genome served as the reference framework for all bioinformatic comparisons and subsequent in silico docking simulations conducted in this study.

#### Enzyme datasets

The 3D structures of the enzymes selected as candidates for molecular docking simulations were retrieved from the Protein Data Bank (PDB) and are listed in Table 1S in the supplementary information. These candidate enzymes included: endospore coat protein from *B. subtilis*, arylsulfatase from *Pseudomonas aeruginosa*, methyl transferase from *Thermotoga maritima*, hydrolase from *B. subtilis*, peptidoglycan-binding protein from *B. subtilis*, xenobiotic oxidoreductase from *Pseudomonas putida*, flavoprotein monooxygenase from *Klebsiella pneumoniae*, CotA laccase from *B. subtilis*, nitro reductase from *B. subtilis*, phosphatase from *B. subtilis*, Cytochrome P450 from *B. subtilis* and peroxygenase from *B. subtilis*, with codes 1GSK, 1HDH, 3BQ5, 3C7E, 3D30, 3L65, 3RP8, 3ZDW, 32SN, 4ETI, 7WYG, 8HKD respectively (Berman et al. 2003).

These enzymes were primarily proposed for their functional relevance to hydrolysis, oxidation, and dehalogenation reactions, which can be involved in the degradation of organochlorine, organophosphate, and carbamate pesticides, including lindane, parathion, and chlorpyrifos, carbaryl, and other halogenated cyclodienes (Sarker et al. 2021). In some cases, enzymes with catalytic promiscuity were also included to represent broader metabolic flexibility within *B. subtilis*. In other words, this concept refers to an enzyme’s ability to catalyze secondary, or “non-native”, reactions in addition to its primary, evolutionarily selected functions (Tawfik et al. 2010).

Additionally, to ensure biological relevance, the sequences of all enzymes were compared to the annotated *B. subtilis* sp. UAMC genome using BLASTp (Johnson et al. 2008). This validation step ensured that the selected structures were genetically and functionally aligned with the metabolic potential of *B. subtilis* sp. UAMC.

#### Enzyme preparation

Retrieved enzymes were prepared using USCF Chimera 1.18 software. Preparation involved removing water molecules, adding hydrogen atoms, and eliminating unwanted ligands from the original receptor structure (Pettersen et al. 2004).

#### Metabolite datasets

The metabolite structures for this study were obtained from the National Center for Biotechnology Information PubChem Compound summary (NCBI 2023a; NCBI 2023b; NCBI 2023c; NCBI 2023d).

#### Metabolite preparation

Ligands were prepared and optimized using UCSF Chimera 1.18 software (Pettersen et al. 2004). Initially, all unwanted residues were removed, then hydrogen atoms were added. The energy minimization used the Steepest Descent algorithm with a convergence threshold of 0.02 Å to ensure structural stability.

#### Molecular docking simulation

The molecular simulations were performed using AutoDock Vina 1.2.2 software (Eberhardt et al. 2021). The docking grid box was adjusted to ensure full protein coverage. The simulation parameters were an exhaustiveness value of 8, which trades off between accuracy and computing time. Additionally, the docking modes were also determined to 9 to obtain multiple ligand conformations. Next, an output file containing the most favorable ligand-receptor conformations was analyzed using UCSF Chimera to evaluate molecular interactions at the active site, including hydrogen bonds and hydrophobic interactions (Pettersen et al. 2004). The docking conformations generated by AutoDock and the related molecular interactions were analyzed using Discovery Studio Visualizer v25.1.0.24284 to identify key binding residues and interaction modes (Besemer et al. 2005). The results were evaluated based on the binding free energy of the generated conformations, with the lowest energy value being selected as the most energetically favorable.

**Fig. 1:**
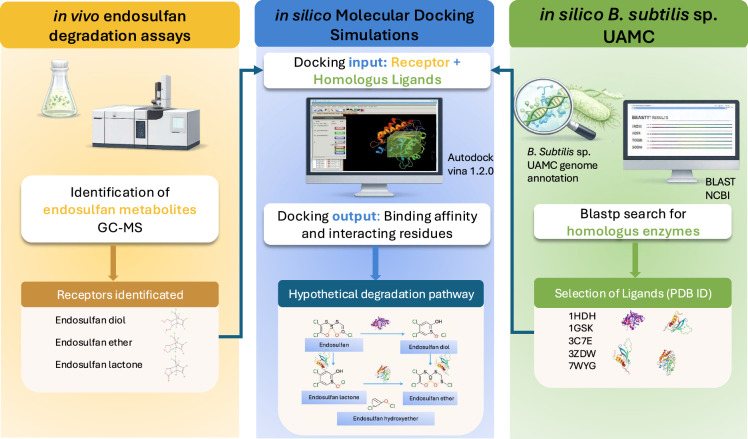
Study design for proposing a hypothetical degradation pathway

To ensure the biological and physical accuracy of our model, the proposed degradation pathway was built upon three concurrent criteria: (i) genomic homology and prioritizing modeled enzymes that share > 98% of sequence identity with the *B. subtilis* sp. UAMC genome; (ii) thermodynamic affinity, to ensure that the selected enzyme-ligand complex exhibits the most negative binding free energy, and (iii) structural viability to verify the formation of key catalytic interactions, such as hydrogen and halogen bonds. The integrated workflow for the endosulfan degradation pathway is described in Fig. 1.

### Data analysis

The Gompertz sigmoidal model was used to fit CO_2_ production data for *B. subtilis* sp. UAMC and calculate its maximum productivity (Casanova et al. 2021; Tjørve et al. 2017). The model could be described by the following equation:

$$y\left(x\right)=a\bullet {e}^{{-e}^{-k(x-{x}_{c})}}$$

where *y* is the cumulative CO_2_ production (mg), *x* refers to the incubation time, a is the maximum asymptotic CO_2_ production (mg), *x*_*c*_ is the time at which the inflection point of the curve occurs, and *k* is the specific CO_2_ production rate (time^−1^).

One-way analysis of variance (ANOVA) with a 95% confidence level and post hoc (Tukey) tests were performed in the IBM SPSS 27 software to assess differences between tests and controls.

## Results and discussion

### in vivo results

#### Degradation of endosulfan by *B. subtilis* sp. UAMC and biomass production

**Fig. 2:**
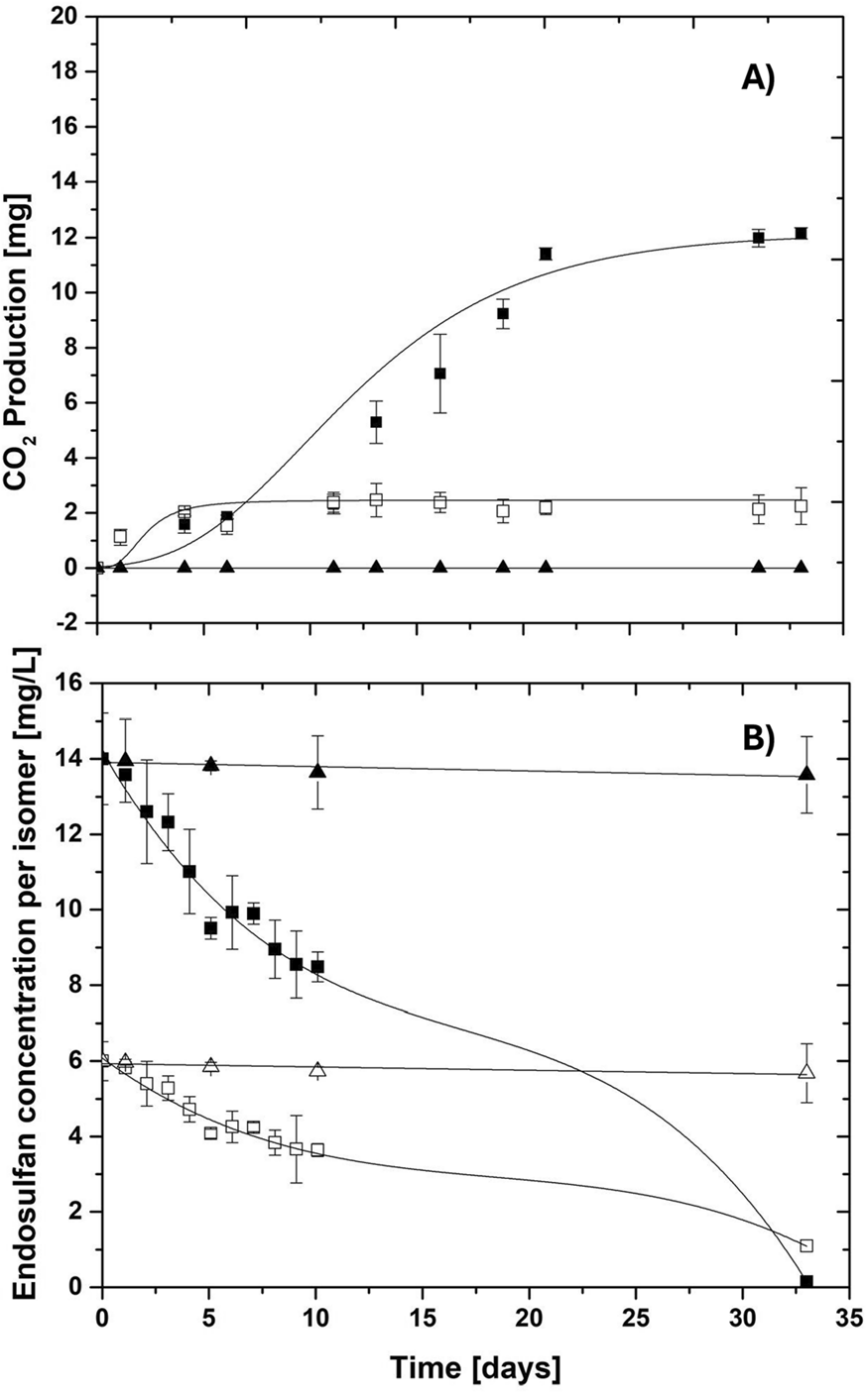
**A** CO_2_ production by *B. subtilis sp*. UAMC. (▲) abiotic control, (□) endogenous control, and (■) experiment. The trend line corresponds to the Gompertz model's data fit: a = 12.154 mg, xc = 9.69 days, and k = 0.1795 d^−1^. **B** Endosulfan isomers degradation. α-endosulfan (■), α-endosulfan control (▲),β-endosulfan (□) and β-endosulfan control (△)

The activity of *B. subtilis* sp. UAMC was evaluated over 33 days, as shown in Fig. 2A. Each test was conducted with its respective endogenous control (in the absence of a carbon source) and an abiotic control (in the absence of bacterial culture). The degradation kinetics showed a rapid adaptation of approximately 4 days (100 h), which correlates with the adaptation of the strain to the medium. Following this period, an exponential increase in CO_2_ release was observed, indicating active metabolism and mineralization of the degradation byproducts. The peak of this metabolic activity occurred in the mid-exponential phase, around 12 days (300 h), and then stabilized as the substrate was depleted. The maximum CO_2_ production reached 12.15 ± 3.98 mg, with a sustained production rate of 0.576 ± 0.168 mg/day for nearly 21 days.

Similar dynamics have been observed in other pesticide-degrading bacterial systems, such as those targeting glyphosate, atrazine, and chlorpyrifos, supporting the hypothesis that *B. subtilis* sp. UAMC expresses inducible metabolic pathways capable of assimilating endosulfan (Góngora-Echeverría et al. 2019; Yadav et al. 2024). In the endogenous control, low measurable CO_2_ was detected, while biomass remained nearly constant. This indicates that *B. subtilis* sp. UAMC maintains basal metabolic activity under carbon-limiting conditions by entering a stationary state of metabolic maintenance (Fig. 2A). The ability to modulate metabolic rates and persist in nutrient-poor environments is a known feature of *Bacillus* spp., allowing them to survive long periods of stress before triggering high-energy processes such as sporulation (Abid et al. 2025; Gray et al. 2019).

The degradation profiles of α-endosulfan and β-endosulfan, shown in Fig. 2B, revealed a gradual increase in biodegradation during the first 10 days of incubation. The experiment started with a total endosulfan concentration of 20 mg/L, which consisted of 14 mg/L of α-endosulfan and 6 mg/L of β-endosulfan. Reliable within these initial conditions, the temporal analysis showed that within the first day, overall degradation reached 3% ± 0.01, progressing to 39.40% ± 0.56 by 10 days, indicating gradual adaptation of *B. subtilis* sp. UAMC to the xenobiotic compounds.

As incubation continued, this trend became more pronounced, and after day one, a clear difference between isomers became evident: α-endosulfan reached 97.60% ± 1.15, whereas β-endosulfan reached 69.45% ± 5.75. This highlights the potential influence of physicochemical factors such as molecular stability and steric accessibility on microbial susceptibility. These findings are similar to previous reports, in which α-endosulfan was more easily biodegraded due to its lower environmental stability and higher bioavailability (Elsaid et al. 2010; Mir et al. 2017).

Calculated degradation rates were 0.16 mg/L/day for α-endosulfan and 0.135 mg/L/day for β-endosulfan. These are higher than those reported for previously described fungal and bacterial strains under comparable conditions. For example, the fungus *Purpureocillium lilacinum* and the bacterium *Sphingobacterium* sp. were reported to degrade α-endosulfan at rates of only 0.06 mg/L/day and 0.089 mg/L/day, respectively, even after incubation periods of up to 40 days, during which only limited degradation was achieved (Hernández-Ramos et al. 2019). These comparisons highlight that the UAMC strain can transform endosulfan more efficiently and within a shorter timeframe.

A similar trend is observed when comparing *B. subtilis* sp. UAMC with other *Bacillus* species. For instance, *Bacillus* sp. PRB77 showed a much lower degradation rate of 0.042 mg/kg/day in soil, and importantly, this activity depends on the addition of dextrose as an external carbon source (Rani et al. 2017). In contrast, the performance of *B. subtilis* sp. UAMC suggests a greater degree of metabolic independence, a particularly valuable trait for bioremediation since it reduces the need for nutrient supplementation and may simplify field application. This is relevant from an applied perspective and will be discussed in greater detail in the following section.

Nevertheless, some gram-negative bacteria, especially strains of *Pseudomonas aeruginosa*, have been reported to degrade endosulfan at higher rates. For example, a degradation rate of 26.4 mg/L/day was reported when endosulfan was used as the sole carbon source (Thangadurai et al. 2014). In another study, a rate of 8.85 mg/L/day was reported for a strain isolated from a cockroach microbiome (Ozdal et al. 2016). These values appear far superior to those observed for *B. subtilis* sp. UAMC. However, such comparisons should be interpreted carefully, since those studies were conducted with a much higher initial endosulfan concentration (100 mg/L), whereas the present study was conducted at 20 mg/L. Because degradation rates are strongly influenced by substrate availability, higher starting concentrations often lead to higher daily mass removal rates.

The differences observed in the biodegradation efficiency between the α- and β-endosulfan isomers suggested a stereoselective behavior of the microbial enzymatic systems involved. This type of isomer-specific biodegradation is relevant in environmental bioremediation, since the stereochemical configuration of a pesticide can strongly influence how easily it interacts with and binds to the active sites of degrading enzymes (Hu et al. 2022). This structural dependency aligns with our in silico predictions, suggesting that the initial catalytic steps are highly sensitive to isomeric conformations of endosulfan.

On the other hand, abiotic controls recovered 72.67% ± 17.76, indicating a 27.3% loss of the pesticide. This reduction could be related to non-biological processes such as volatilization, photodegradation, or adsorption to the surfaces of the experimental system. These abiotic losses were accounted for in the calculation of net biodegradation to avoid overestimating microbial activity (Yasir et al. 2025).

The experimental conditions yieled a net concentration of biomass of 30.833 ± 0.001 mg/L, indicating that *B. subtilis* sp. UAMC mineralizes endosulfan and assimilates its carbon into cellular components. These experimental results align with our in silico findings, where we identified enzymes capable of degrading endosulfan.

The in silico analysis identified interactions between some enzymes, such as hydrolases (*e.g*., 3C7E), cytochrome P450 oxidoreductases (*e.g*., 7WYG), and CotA laccase (e.g., 3ZDW), and showed they could interact with endosulfan in our docking analysis. This connection proved that the strain possessed the necessary enzymatic tools to mineralize the compound without accumulating toxic residues (Ebsa et al. 2024; Swathy et al. 2024). All these findings can suggest the role of these enzymes in the endosulfan degradation.

In the next section, we analyzed the transformation products generated during the process.

#### Metabolite identification

**Fig. 3:**
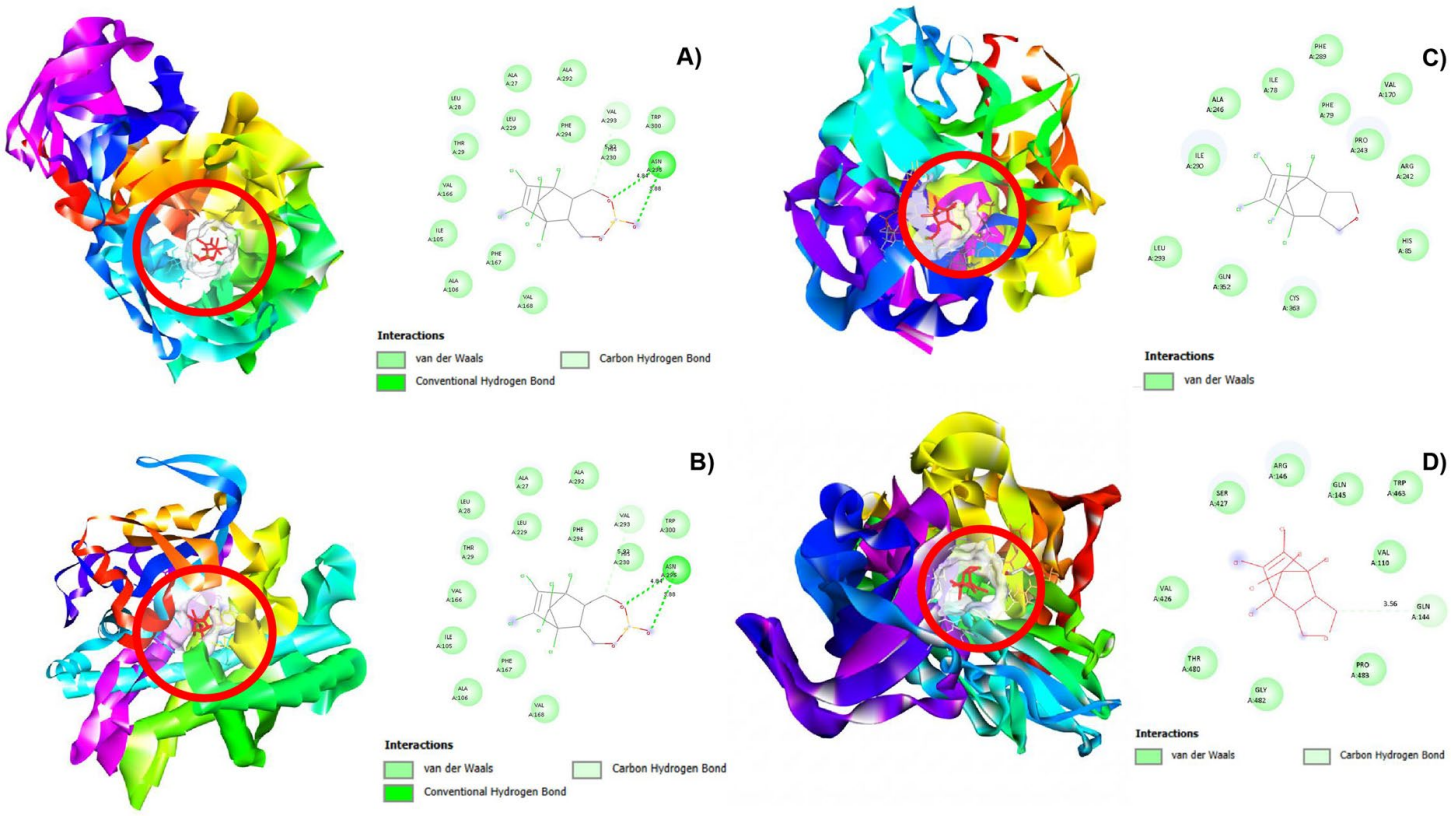
Interactions between endosulfan and its metabolites with the enzymes by molecular docking. The enzyme-binding sites are represented as solid surfaces coloured in white. The enzymes binding sites are represented as solid surfaces using BIOVIA Discovery Studio to visualize specific residues and bonding types: **A** endosulfan bound within the active site of enzyme 3C7E; **B** interaction of endosulfan diol at the catalytic pocket of enzyme 3ZDW; **C** binding of endosulfan ether to the active site of enzyme 7WYG, and **D** interaction of endosulfan hydroxyether within the active site of enzyme 1GSK. In each case, the metabolite is docked in the enzyme’s active site

**Fig. 4:**
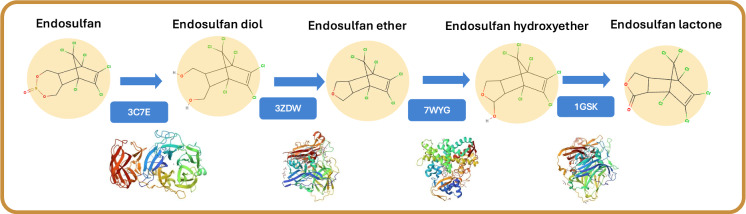
Hypothetical pathway proposed for endosulfan degradation by *B. subtilis* sp. UAMC

The degradation products identified during the biodegradation assays are summarized in Table 1. Gas chromatography-mass spectrometry (GC–MS) analysis revealed five metabolites with retention times (RT) ranging from 10.37 to 27.35 min. The mass spectra of each metabolite is available in the Supplementary material (Figs. 1S to 5S).

**Table 1:** Identified metabolites in degradation experiments

RT^a^ (min)	Compound	Formula	#CAS	Quality (%)
10.37	Azulene, 2,2,3,4,5,6,7- octahydro-1,4 dimethyl-7	C_15_H_24_	22567-17-5	90
19.84	1,2 Benzenedicarboxylic acid bis (2 methylpropyl) ester	C_16_H22O4	084-69-5	90
20.15	4,7-methanoisobenzofuran, 4,5,6,7,8,8 hexachloro-1,3, 3a,4,7,7a-hexahydro- (endosulfan ether)	C_9_H_6_Cl_6_O	3369-52-6	92
27.32	4,7-methanoisobenzofuran- 1[3H]-one, 4,5,6,7,8,8- hexachloro-3a,4,7, 7tetrahydro- (endosulfan lactone)	C_9_H_4_Cl_6_O_2_	3868-61-9	90
27.35	Bicyclo (2.2.1) hept-5-ene-2,3-dimethanol, 1,4,5,6,7,7 hexachloro- (endosulfan diol)	C_9_H_8_Cl_6_O_2_	2157–19-9	86

^a^Retention time

Three of these metabolites, endosulfan ether, endosulfan lactone, and endosulfan diol, were detected exclusively under biotic conditions and were absent in abiotic controls. This is important because endosulfan diol can also be formed by spontaneous hydrolysis of the pesticide under alkaline conditions (Sutherland et al. 2002). Therefore, its absence in the uninoculated controls provides strong evidence that the metabolites identified in this study were generated through biological activity rather than by abiotic transformation.

The detection of these metabolites, together with the absence of endosulfan sulfate, strongly suggests that *B. subtilis* sp. UAMC follows a non-toxic, hydrolytic pathway (Deng et al. 2016). In environmental systems, microbial degradation of endosulfan generally proceeds through two main routes: oxidative or hydrolytic. The oxidative one, described in certain *Mycobacterium* strains, involves enzymes such as ESD monooxygenase and can convert endosulfan into endosulfan sulfate, a metabolite known to be more toxic and environmentally persistent than the parent compound (Kataoka et al. 2013; Sutherland et al. 2002). In contrast, the hydrolytic pathway, as reported in bacteria such as *Pseudomonas aeruginosa*, *Burkholderia cepacia,* and *Pseudomonas fluorescens,* produces the less toxic metabolite endosulfan diol, which may subsequently be transformed into endosulfan ether or hydroxyether, and later into endosulfan lactone (Awasthi et al. 2003; Hussain et al. 2007; Jesitha et al. 2015; Kumar et al. 2006b; Sathishkumar et al. 2021).

Furthermore, the hydrolytic mechanism exhibited by *B. subtilis* sp. UAMC is environmentally advantageous because it appears to avoid the buildup of toxic oxidative residues and produces more polar, biodegradable ones (Sathishkumar et al. 2021). These findings, obtained under controlled laboratory conditions and using pure cultures, complement the previously reported degradation kinetics, which confirm that endosulfan serves as the sole carbon source for *B. subtilis* sp. UAMC (Mitra et al. 2024).

To fully understand the bioremediation potential of *B. subtilis* sp. UAMC, it is important to place its performance in context by comparing it with other microorganisms previously reported as capable of degrading endosulfan. Strains including *Staphylococcus* sp., *Bacillus circulans*, *Bacillus* sp., and plant-growth-promoting rhizobacteria (PGPR) have achieved meaningful degradation rates only when an external carbon source, typically dextrose, was added to the growth medium (Kumar et al. 2006a; Rani et al. 2017). Similarly, certain *Stenotrophomonas* and *Arthrobacter* species appear to transform endosulfan co-metabolically, meaning the pesticide is broken down as a byproduct of other metabolic processes rather than being deliberately exploited as a nutritional resource (Sutherland et al. 2000). Against this, the ability of *B. subtilis* sp. UAMC to use endosulfan as its sole carbon and energy source could reflect a more direct, highly efficient metabolic degradation strategy.

In addition to the degradation intermediates, two bioactive metabolites were identified: azulene, 2,2,3,4,5,6,7-octahydro-1,4-dimethyl-7, and bis(2-methylpropyl) benzene-1,2-dicarboxylate. Azulene is a bicyclic sesquiterpene derivative with recognized insecticidal activity, previously isolated from *Eucalyptus citridora* extracts and effective against *Tribolium castenum* (Sahi 2016). The second one, bis (2-methylpropyl) benzene-1,2-dicarboxylate, has been described as an antimicrobial compound naturally synthesized by *B. subtilis* (Koilybayeva et al. 2023). Its detection reinforces the hypothesis that, in response to environmental stressors such as xenobiotic compounds, *B. subtilis* sp. UAMC activates secondary metabolic pathways, simultaneously promoting the degradation and biosynthesis of bioactive protective molecules (Mitra et al. 2024).

These results provide the experimental basis for exploring the previously presented biodegradation pathways, and molecular docking analyses clarify the potential enzymatic interactions responsible for their transformation.

### In silico analytical results

#### Molecular docking and endosulfan hypothetical degradation pathway.

Comparative BLASTp analysis of the *B. subtilis* sp. UAMC genome indicated that most candidate enzymes retrieved from the Protein Data Bank shared high sequence identity (> 95%) with their corresponding genomic homologs, confirming their physiological relevance in the strain (Table 2). While enzymes lacking direct high homology, such as 1HDH and 3L65, demonstrated an identity percentage of 40.54% and 39.33%, respectively were initially evaluated due to general catalytic resemblances, the construction of the final proposed degradation pathway strictly prioritized enzymes with near complete genomic identity (e.g., 3d30 with 100% identity and 1GSK, 3C7E, 3ZDW and 7WYG with > 98% identity). This stringent selection ensures the highest biological and physiological accuracy for our model without the immediate need for transcriptomic assays (Letizia et al. 2025; Tuon et al. 2022).

**Table 2:** BLASTp alignment data for the subset of enzymes *B. subtilis* sp. UAMC

PDB ID	Closest CDS in *B. subtilis* sp. UAMC	Identity %	Query cover %	E-value	Number of gap openings
1GSK	CDS_1901	99.53	100	0	521
1HDH	CDS_3216	40.54	7	0.005	653
3BQ5	CDS_1487	95	95	0	762
3C7E	CDS_2006	98.36	100	0	513
3D30	CDS_2055	100	100	3e-156	232
3L65	CDS_2403	39.33	98	2e-73	338
3RP8	CDS_2315	65.52	7	0.001	174
3ZDW	CDS_3119	99.42	100	0	513
3N2S	CDS_288	99.60	100	0	249
4ETI	CDS_409	98	82	2e-109	156
7WYG	CDS_3664	98.082	100	0	417
8HKD	CDS_3664	97.84	98	0	417

Molecular docking overview. The molecular docking results showed that *B. subtilis* sp. UAMC’s capability to mineralize endosulfan does not depend on a unique specialized degradation pathway. Otherwise, it could depend on the catalytic promiscuity of endogenous enzymes acting together in a synergetic consortium (Tawfik et al. 2010). These results allow enzymes with evolutionarily primary functions to be selected, such as the CotA laccase (1GSK/3ZDW) or Cytochrome p450 (7WYG), and specific hydrolases (3C7E, 3D30), to keep sufficient structural plasticity to recognize and transform nonnative xenobiotic substrates (Enguita et al. 2003; Kammoonah et al. 2018). The favourable binding energies found ranging from − 5.7 to − 7.6 kcal/mol could indicate that the active sites of these enzymes possess sufficient structural plasticity to stabilize this type of chlorinated metabolites, which could facilitate dehydration, hydroxylation, and cyclization reactions that, although secondary for the enzymes, are critical for pesticide detoxification which could suggest spontaneous and stable interactions consistent with catalytic feasibility (Rojmala et al. 2024; Zhang et al. 2024). Visual inspection of the binding poses confirmed that each metabolite was properly positioned within the catalytic pocket of its respective enzyme, stabilized through a combination of hydrogen bonds, van der Waals forces, and hydrophobic interactions, as follows in Fig. 3. The amino acid residues identified align with catalytic motifs typical of hydrolases and redox enzymes, supporting the accuracy of the molecular docking simulations and the feasibility of these promiscuous enzymes.

The distances between key atoms in these interactions were also evaluated to assess the stability and specificity of the binding event, as detailed in Table 3, which contains information solely on the enzymes whose reactions were implicated in the proposed endosulfan degradation pathway described here. Details on the other enzymes tested for docking analyses are provided in the supplementary information, Table 2S.

**Table 3:** Binding energy, residues, and distances estimated by molecular docking calculation

Enzyme	Susbtrate	Binding energy (kcal/mol)	Residue	Distances (Å)
1GSK	ENDOSULFAN	− 6.6	Gln 362ᵃ	
			Trp 463ᵃ	
			Gly 482ᵃ	
			Val 426ᵃ	
			Pro 483ᵃ	
			Gol 1512ᵃ	
			Val 110ᵃ	
			Gln 145ᵃ	
			Arg 146ᵇ	3.45
			Arg146ᵇ	4.63
			Ser 427ᶜ	4.57
			Thr 480ᶜ	3.62
			Gln 144ᶜ	6.87
1GSK	ENDOSULFAN DIOL	− 5.9	Trp 463ᵃ	
			Pro 483ᵃ	
			Thr 480ᵃ	
			Arg 146ᵃ	
			Gln 144ᵃ	
			Gol 1512ᵃ	
			Arg 64ᵃ	
			Gln 145ᵃ	
			Val 110ᵃ	
			Gly 482ᵃ	
			Val 426ᵃ	
			Gln 362ᵇ	5.46
			Ser 427ᵇ	2.91
			Ser 427ᵈ	3.18
	ENDOSULFAN ETHER	− 6	Ser 427ᵃ	
			Arg 146ᵃ	
			Gln 145ᵃ	
			Trp 463ᵃ	
			Val 110ᵃ	
			Pro 483ᵃ	
			Gly 482ᵃ	
			Thr 480ᵃ	
			Val 426ᵃ	
			Gln 144ᶜ	6.44
	ENDOSULFAN HYDROXYETHER	− 6	Ser 427ᵃ	
			Arg 146ᵃ	
			Gln 145ᵃ	
			Trp 463ᵃ	
			Val 110ᵃ	
			Pro 483ᵃ	
			Gly 482ᵃ	
			Thr 480ᵃ	
			Val 426ᵃ	
			Gln 144ᵇ	6.41
	ENDOSULFAN LACTONE	− 6.6	Ser 427ᵃ	
			Arg 146ᵃ	
			Gln 145ᵃ	
			Trp 463ᵃ	
			Val 110ᵃ	
			Pro 483ᵃ	
			Gly 482ᵃ	
			Thr 480ᵃ	
			Val 426ᵃ	
			Gln 144ᵇ	6.44
3C7E	ENDOSULFAN	− 7.6	Leu 28ᵃ	
			Ala 27ᵃ	
			Ala 292ᵃ	
			Leu229ᵃ	
			Phe 294ᵃ	
			Trp 300ᵃ	
			Val 167ᵃ	
			Phe 167ᵃ	
			Ala 106ᵃ	
			Ile 105ᵃ	
			Val 166ᵃ	
			Thr 29ᵃ	
			Asn 295ᵇ	4.84
			Asn 295ᵇ	3.88
			Val 293ᶜ	5.92
	ENDOSULFAN DIOL	− 6.6	Val 166ᵃ	
			Ile 105ᵃ	
			Leu 229ᵃ	
			Ala 106ᵃ	
			Phe 167ᵃ	
			Lys 231ᵃ	
			His 230ᵃ	
			Trp 300ᵃ	
			Val 293ᵃ	
			Ala 27ᵃ	
			Thr 29ᵃ	
			Leu 28ᵃ	
			Asn 295ᵇ	4.81
			Asn 295ᵇ	4.43
			Val 168ᶠ	4.28
	ENDOSULFAN ETHER	− 6.7	Leu 28ᵃ	
			Ile 105ᵃ	
			Phe 294ᵃ	
			Asn 295ᵃ	
			Trp 300ᵃ	
			Val 293ᵃ	
			Lys 231ᵃ	
			His 230ᵃ	
			Leu 229ᵃ	
			Phe 167ᵃ	
			Val 166ᵃ	
			Ala 292ᵃ	
			Val 168ᵃ	
			Ala 27ᵃ	
			Thr 29ᵃ	
	ENDOSULFAN HYDROXYETHER	− 6.7	Leu 28ᵃ	
			Ile 105ᵃ	
			Phe 294ᵃ	
			Asn 295ᵃ	
			Trp 300ᵃ	
			Val 293ᵃ	
			Lys 231ᵃ	
			His 230ᵃ	
			Leu 229ᵃ	
			Phe 167ᵃ	
			Val 166ᵃ	
			Ala 292ᵃ	
			Val 168ᵃ	
			Ala 27ᵃ	
			Thr 29ᵃ	
	ENDOSULFAN LACTONE	− 7	Ala 27ᵃ	
			Thr 29ᵃ	
			Ile 105ᵃ	
			Ala 106ᵃ	
			Trp 300ᵃ	
			Lys 231ᵃ	
			Phe 167ᵃ	
			Val 293ᵃ	
			Val 166ᵃ	
			Val 168ᵃ	
			Ala 292ᵃ	
			Leu 28ᵃ	
			Asn 295ᵇ	4.36
			Phe 294ᶜ	
			Leu 229ᶜ	6.37
3ZDW	ENDOSULFAN	− 6.6	Phe 228ᵃ	
			Val 225ᵃ	
			Ala 211ᵃ	
			Pro 212ᵃ	
			Pro 209ᵃ	
			Ala 227ᵃ	
			Glu 213ᵉ	6.71
			Cys 229ᵇ	4.35
			Pro 226ᶜ	4.48
	ENDOSULFAN DIOL	− 5.7	Pro 384ᵃ	
			Leu 386ᵃ	
			Ala375ᵃ	
			Gln 442ᵃ	
			Arg 416ᵃ	
			Val 385ᵇ	4.86
			Thr 377ᵇ	4.73
			Gly 376ᵇ	3.2
			Thr 415ᶜ	4.03
	ENDOSULFAN ETHER	− 5.5	Glu 213ᵃ	
			Ala 211ᵃ	
			Pro 212ᵃ	
			Pro 209ᵃ	
			Val 225ᵃ	
			Phe 228ᵃ	
			Ala 227ᵃ	
			Pro 226ᶜ	4.4
			Cys 229ᶠ	3.96
	ENDOSULFAN HYDROXYETHER	− 5.5	Cys 229ᵃ	
			Ala 227ᵃ	
			Glu 213ᵃ	
			Ala 211ᵃ	
			Pro 212ᵃ	
			Pro 209ᵃ	
			Phe 228ᵃ	
			Val 225ᵃ	
			Pro 226ᶜ	4.45
	ENDOSULFAN LACTONE	− 6	Pro 209ᵃ	
			Pro 212ᵃ	
			Glu 213ᵃ	
			Gly 323ᵃ	
			Ala 227ᵃ	
			Val 225ᵃ	
			Cys 229ᵇ	3.45
			Pro 226ᵇ	3.98
7WYG	ENDOSULFAN	− 6.6	Phe 289ᵃ	
			Gln 352ᵃ	
			Cys 363ᵃ	
			His 361ᵃ	
			Phe 79ᵃ	
			Ile 78ᵃ	
			Ala 246ᵃ	
			Arg 242ᵃ	
			Pro 243ᵃ	
			His 85ᵈ	6.28
	ENDOSULFAN DIOL	− 5.1	Phe 289ᵃ	
			Ile 290ᵃ	
			Gln 352ᵃ	
			Cys 363ᵃ	
			Pro 364ᵃ	
			Gly 365ᵃ	
			His 85ᵃ	
			Arg 242ᵃ	
			Val 170ᵃ	
			Pro 243ᵃ	
			Phe 79ᵃ	
			Ala 246ᵃ	
			Ile 78ᵃ	
			Leu 293ᵃ	
	ENDOSULFAN ETHER	− 6.6	Ala 246ᵃ	
			Ile 78ᵃ	
			Phe 289ᵃ	
			Val 170ᵃ	
			Pro 243ᵃ	
			Arg 242ᵃ	
			Arg 242ᵃ	
			His 85ᵃ	
			Cys 363ᵃ	
			Gln 352ᵃ	
			Leu 293ᵃ	
			Ile 290ᵃ	
	ENDOSULFAN HYDROXYETHER	− 6.6	Phe 289ᵃ	
			Val 170ᵃ	
			Phe 79ᵃ	
			Val 170ᵃ	
			Pro 243ᵃ	
			Arg 242ᵃ	
			His 85ᵃ	
			Cys 363ᵃ	
			Gln 352ᵃ	
			Leu 293ᵃ	
			Ile 290ᵃ	
			Ala 246ᵃ	
			Ile 78ᵃ	
	ENDOSULFAN LACTONE	− 5.7	Ser 273ᵃ	
			Lys 109ᵃ	
			Lys 109ᵃ	
			Glu 371ᵃ	
			Lys 374ᵃ	
			Ala 375ᵃ	
			Asp 378ᵃ	
			Thr 116ᵃ	
			Glu 113ᵃ	
			Ala 112ᶠ	3.79

Types of residues: ^a^van der Waals, ^b^Conventional Hydrogen Bond, ^c^Carbon Hydrogen Bond, ^d^Unfavorable aceptor-aceptor, ᵉAttractive charge, ᶠHalogen

#### Endosulfan to endosulfan diol

The interaction between endosulfan and the hydrolase 3C7E showed the highest binding affinity among all the enzyme–substrate complexes evaluated in this study (− 7.6 kcal/mol). Although 3C7E is typically classified as a glycosyl hydrolase, our in silico thermodynamic analysis, together with genomic alignment (> 98% identity), suggests that this enzyme is more versatile than previously thought. It likely takes advantage of its structural flexibility and catalytic promiscuity to break down the otherwise resistant sulfite ester bond. A closer look at the structure of the complex indicates it is anchored mainly by two conventional hydrogen bonds with Asn 295 (3.88 Å and 4.84 Å). These relatively short distances suggested a meaningful interaction, indicating that this residue may play an important role in stabilizing the substrate and promoting the bond polarization needed to initiate hydrolysis (Wei et al. 2024).

At the same time, the bulky and highly lipophilic hexachlorocyclopentadiene ring is comfortably accommodated within a large hydrophobic pocket. This positioning is supported by a carbon-hydrogen interaction with Val 293 (5.92 Å), along with an extensive network of van der Waals interactions involving mostly nonpolar residues such as Leu 28, Ala 27, Ala 292, His 230, Leu 229, Phe 294, Trp 300, Thr 29, among others. Together, these interactions help stabilize and properly orient the bulky chlorinated ring, placing it in an optimal position for a nucleophilic attack (Saribas et al. 2012; Zhao et al. 2020). This specific arrangement within the active site appears to create favorable conditions for the initial hydrolytic cleavage of the sulfite ring, consistent with the experimental detection of endosulfan diol by GC–MS.

It is also important to mention that the absence of an oxidative environment within these binding sites explains why the pathway bypasses the formation of toxic endosulfan sulfate, a key detoxifying trait of *B. subtilis* sp. UAMC (Blanco-Míguez et al. 2023; Shebis et al. 2022).

#### Endosulfan diol to endosulfan ether

While the transformation of endosulfan to endosulfan diol is widely accepted as a hydrolytic step, the subsequent cyclization to endosulfan ether has been far less explored. Although cytochrome P450 and monooxygenases are often involved in similar pathways, in this study, the multicopper oxidase CotA, represented here by the highly homologous structure 3ZDW (over 99% genomic identity), is the most likely candidate driving this transformation (Singh et al. 2019; Ulcnik et al. 2013). The enzymes T1, T2, and T3 copper centers appear to facilitate the dehydration of endosulfan diol via a promiscuous redox mechanism (Gabdulkhakov et al. 2019).

In our sequential model, molecular docking indicated that 3ZDW has a strong and specific binding affinity for endosulfan diol (− 5.7 kcal/mol). The interaction does not seem to depend solely on general hydrophobic contacts; instead, the substrate appears to be carefully positioned toward the catalytic region through a network of three conventional hydrogen bonds involving Gly 376 (3.20 Å), Thr 377 (4.73 Å), and Val 385 (4.86 Å). This polar anchoring suggests a strong polarizing pull on the exposed hydroxyl groups of the diol. This arrangement is complemented by a carbon-hydrogen bond with Thr 415 (4.03 Å) and a surrounding hydrophobic pocket formed by residues such as Pro 384, Leu 386, and Ala 375, which accommodates the bulky organochlorine structure of the substrate. Altogether, this combination of interactions appears to stabilize the transition state, facilitating processes such as proton abstraction and water removal. In this way, endosulfan diol may be directed toward etherification, likely supported by the catalytic flexibility of the enzyme (Augustine et al. 2010; García-Hernández et al. 2024; Pekgenc et al. 2022; ur Rahman et al. 2024).

#### Endosulfan ether to endosulfan hydroxyether

Although endosulfan hydroxyether was not directly identified by GC–MS analysis, it was included in the in silico modeling to evaluate the structural continuity of the proposed degradation pathway. The absence of this metabolite in experimental assays is likely due to its role as a short-lived transient intermediate, rapidly channeled toward the formation of endosulfan lactone (Sutherland et al. 2002). To test the feasibility of this oxidative step, molecular docking was analyzed with cytochrome P450 (7WYG).

These simulations strongly supported the involvement of this enzyme as the optimal candidate for this transition, revealing a highly favorable binding energy (− 6.6 kcal/mol). While other enzymes proposed in this work lose steric affinity as the ether intermediate forms, 7WYG binds the substrate stably within a highly hydrophobic active site pocket. This stabilization is driven by extensive van der Waals interactions with Ala 246, Ile 78, Phe 289, Val 170, and Pro 243, and Cys 363. The precise orientation of the substrate near the heme iron center, coordinated by this Cys 363, facilitates the formation of a reactive oxyferryl intermediate. This type of species enables the hydroxylation of the ether framework, consistent with the P450 enzyme mechanism observed in other organochlorine-degrading bacteria (Jurghen 2019; Kermasha et al. 2021). The favorable thermodynamic affinity and specific structural coordination suggest that the *B. subtilis* sp. UAMC machinery is theoretically capable of facilitating this oxidative transition as part of its promiscuous metabolic repertoire.

#### Endosulfan hydroxyether to endosulfan lactone

The final step in the proposed endosulfan degradation pathway involves the conversion to endosulfan lactone. While specialized strains like *Mycobacterium* sp. rely on specific monooxygenase Esd to manage these downstream metabolites, this result suggests that *B. subtilis* sp. UAMC employs the enzyme 1GSK to drive this transformation (Sutherland et al. 2004). Also, genomic alignment demonstrated that 1GSK shares 99.53% sequence identity with the corresponding CDS in the strain’s genome (Table 2), providing robust, direct biological validation that this specific genetic machinery is present and highly active in our model organism. Crucially, the natural catalytic function of this glucose-1-dehydrogenase, the NADP + dependent oxidation of cyclic hemiacetals into lactones, perfectly mirrors the chemical transformation required to convert endosulfan hydroxyether into endosulfan lactone. This represents a clear example of enzymatic promiscuity, where the endogenous metabolic machinery of *B. subtilis* sp. UAMC. The docking analysis reveals a highly favorable binding energy (− 6.0 kcal/mol) for endosulfan hydroxyether. The substrate appears to be firmly stabilized within the active site by a hydrophobic cluster formed by residues such as Ser 427, Arg 146, Gln 145, Trp 463, Val 110, Pro 483, Gly 482, Thr 480, and Val 246, which likely contributes to its proper positioning. Rather than being purely structural, this network of interactions appears actively orient the hemiacetal hydroxyether group in a way that favors proton abstraction, thereby facilitating the ring closure required to form a more stable lactone structure (Bakour et al. 2023).

Mechanistically, the hydrophobic microenvironment created by two residues, Trp 463 and Val 110, excludes local water molecules, a condition that thermodynamically favors this dehydration ring closure (Charendoff et al. 2013; Ha et al. 2016; Jiang et al. 2024). Taken together, the strong genomic correspondence and the observed structural features support the idea that 1GSK likely functions as a key catalyst in this final oxidative step within the bacterial enzymatic system.

### Hypothetical pathway proposed

The interaction analyses performed via molecular docking of the in vitro assays, resulted in a proposed hypothetical endosulfan degradation pathway by *B. subtilis* sp. UAMC (Fig. 4). This was based on three complementary levels of evidence: (1) the experimental identification of endosulfan degradation metabolites via GC–MS analysis, (2) enzyme–substrate interaction predictions through molecular docking studies, and (3) comparative homology analysis that confirmed that enzymes modeled from the Protein Data Bank shared > 95% sequence identity with genomic homologs in *B. subtilis* sp. UAMC, as follows in Fig. 1. This multitiered approach enables the formulation of a plausible sequential transformation of endosulfan into endosulfan lactone via intermediate compounds, including endosulfan diol, ether, and hydroxyether, successfully bypassing the formation of the highly toxic endosulfan sulfate.

Although the integration of experimental and in silico data strongly support the proposed degradation sequence, the pathway remains a predictive model. Definitive validation requires isotopic labeling studies to trace metabolic flow and confirm the identity and direction of intermediate formation (Wang et al. 2025). Furthermore, confirming enzyme function through heterologous expression and enzymatic assays will be crucial for verifying the proposed catalytic roles in *B.* *subtilis* sp. UAMC.

Beyond these controlled laboratory findings, the metabolic capabilities and intrinsic biological traits of *B. subtilis* sp. UAMC suggests potential for real-world environmental applications. One of its more valuable characteristics is the natural ability of *B. subtilis* strains to form highly resistant endospores, which provide an important survival advantage under adverse environmental conditions such as temperature, desiccation, and nutrient limitation. This trait makes the strain a particularly promising candidate for direct bioaugmentation at contaminated sites (Checinska et al. 2015; Sarenkova et al. 2026).

In addition, its ability to completely utilize endosulfan without producing a more toxic or recalcitrant compound supports its potential for use in bioreactor systems for treating agricultural runoff or industrial wastewater (Dai et al. 2024; Yadav et al. 2014). Although future soil microcosm and pilot- scale studies will be necessary to assess its performance under more complex environmental conditions and in the presence of native microbial communities, the integration of experimental evidence with the insights presented here highlights the versatility of *B. subtilis* sp. UAMC as a promising tool for environmental bioremediation.

## Conclusion

This study validates the capability of *B. subtilis* sp. UAMC to effectively mineralize endosulfan. While GC–MS analysis confirmed the formation of endosulfan metabolites, such as endosulfan ether, endosulfan diol, and endosulfan lactone, without the presence of the more toxic metabolite endosulfan sulfate. Supported by genomic alignments, our in silico modeling revealed that this process is driven by different enzymes rather than a single pathway. Enzymes such as 3C7E, 3ZDW, 7WYG, and 1GSK utilize their catalytic promiscuity and structural plasticity to sequentially transform the endosulfan intermediates. This synergy between experimental validation and mechanistic insight underscores the versatility of *B. subtilis* sp. UAMC for bioremediation. While combining experimental and in silico data strongly supports the proposed degradation sequence, the pathway remains a predictive model, confirming enzyme function through heterologous expression and enzymatic assays will be crucial for verifying the proposed catalytic roles of the *B. subtilis* sp. UAMC, enzymatic assays and transcriptomic analyses are important for future research directions.

## Supplementary Information

Below is the link to the electronic supplementary material.

Supplementary file1 (DOCX 1346 KB)

## Data Availability

The data supporting this article were included in the supplementary information (SI).
