# Supplementary material for: In vitro and in silico study of the endosulfan degradation by Bacillus subtilis sp. strain UAMC
Source: Biodegradation. 2026 May 19;37(3):89. doi: 10.1007/s10532-026-10312-y (PMC13183696; doi:10.1007/s10532-026-10312-y)
Supplement: Supplementary file 1 — Supplementary file1 (DOCX 1346 KB) [file 10532_2026_10312_MOESM1_ESM.docx]

***In* *vitro* and *in silico* study of the endosulfan degradation by *Bacillus subtilis* sp. strain UAMC**

Adriana Casanova^1^, Sergio Hernández^2^, Diego A. Esquivel-Hernández^2^, Sergio Revah^2^ Irmene Ortíz^2*^

1. Posgrado en Ciencias Naturales e Ingeniería. Universidad Autónoma Metropolitana-Cuajimalpa, Av. Vasco de Quiroga 4871, Col. Santa Fe. C.P. 05348, México City, México.
2. Depto. Procesos y Tecnología. Universidad Autónoma Metropolitana-Cuajimalpa. Av. Vasco de Quiroga 4871, Col. Santa Fe. C.P. 05348, México City, México.

*Corresponding author: Irmene Ortíz; irmene@cua.uam.mx

**Table 1S**. Candidate enzymes retrieved from PDB

| **Enzyme** | **Organism** | **PDB code** |
| --- | --- | --- |
| Endospore coat protein | *Bacillus subtilis* | 1GSK |
| Arylsulfatase | *Pseudomonas aeruginosa* | 1HDH |
| Methyl transferase | *Thermotoga maritima* | 3BQ5 |
| Hydrolase | *Bacillus subtilis* | 3C7E |
| Peptidoglycan-binding protein | *Bacillus subtilis* | 3D30 |
| Xenobiotic oxidorreductase | *Pseudomonas putida* | 3L65 |
| Flavoprotein monooxygenase | *Klebsiella pneumoiniae* | 3RP8 |
| CotA laccase | *Bacillus subtilis* | 3ZDW |
| Nitro reductase | *Bacillus subtilis* | 3N2S |
| Phosphatase | *Bacillus subtilis* | 4ETI |
| Cytochrome P450 | *Bacillus subtilis* | 7WYG |
| Peroxygenase | *Bacillus subtilis* | 8HKD |

**Figure 1S.** Metabolite mass spectrum Azulene, 2,2,3,4,5,6,7- octahydro-1,4 dimethyl-7

**Figure 2S.** Metabolite mass spectrum 1,2 Benzenedicarboxylic acid bis (2 methylpropyl) ester

**Figure 3S.** Metabolite mass spectrum 4,7-methanoisobenzofuran, 4,5,6,7,8,8 hexachloro-1,3, 3a,4,7,7a-hexahydro-


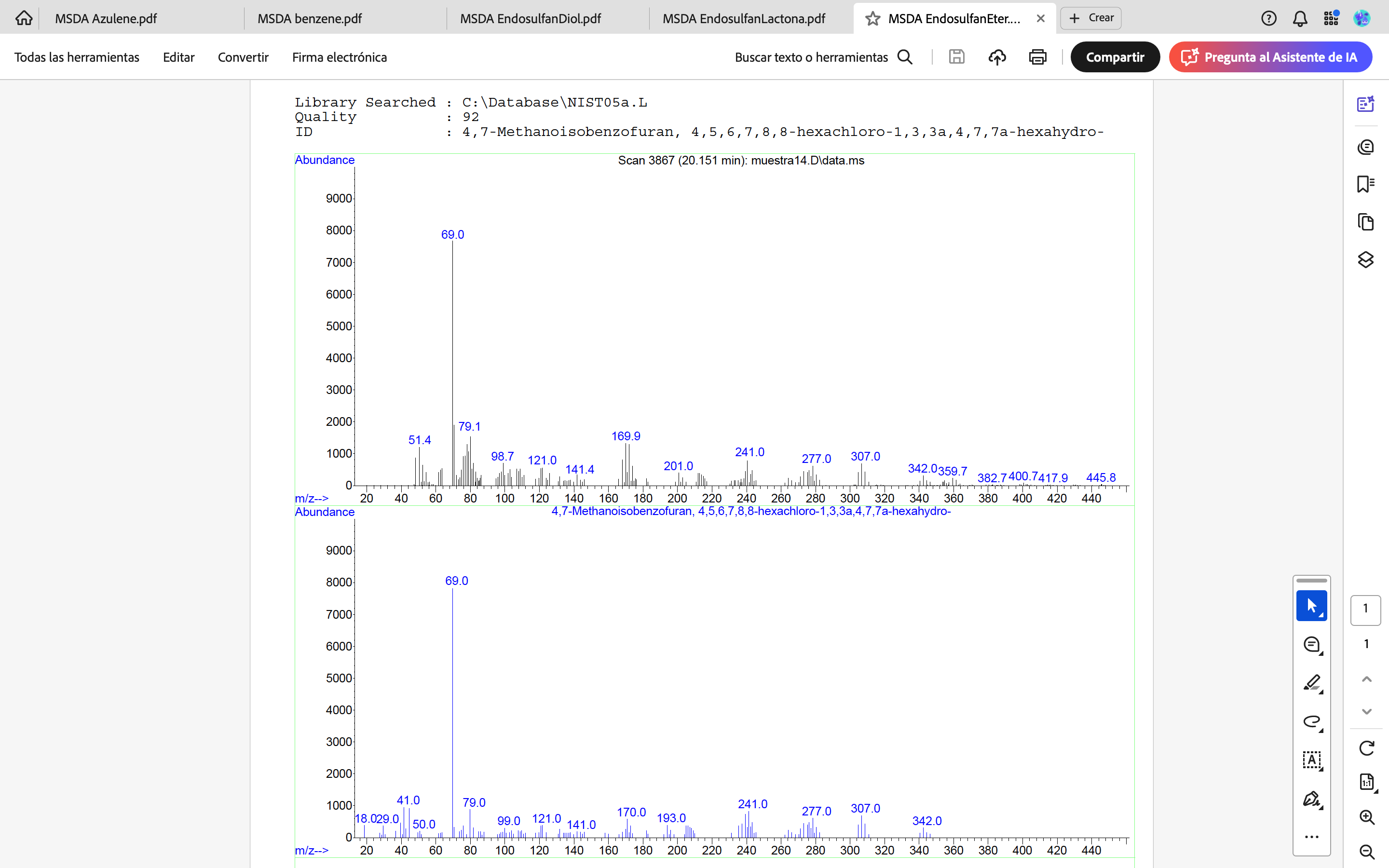


**Figure 4S.** 4,7-methanoisobenzofuran- 1[3H]-one, 4,5,6,7,8,8- hexachloro-3a,4,7, 7tetrahydro-

**Figure 5S.** Bicyclo (2.2.1) hept-5-ene-2,3-dimethanol, 1,4,5,6,7,7 hexachloro-

**Table 2S.** Binding energy, residues, and distances estimated by molecular docking calculation.

| **Enzyme** | **Susbtrate** | **Binding Energy (kcal/ mol)** | **Residue Type** | **Residue** | **Distances (Å)** |
| --- | --- | --- | --- | --- | --- |
| 1GSK | Endosulfan | -6.6 | Van der Waals | Gln 362 |  |
|  |  |  |  | Trp 463 |  |
|  |  |  |  | Gly 482 |  |
|  |  |  |  | Val 426 |  |
|  |  |  |  | Pro 483 |  |
|  |  |  |  | Gol 1512 |  |
|  |  |  |  | Val 110 |  |
|  |  |  |  | Gln 145 |  |
|  |  |  | Conventional Hydrogen Bond | Arg 146 | 3.45 |
|  |  |  |  | Arg146 | 4.63 |
|  |  |  | Carbon Hydrogen Bond | Ser 427 | 4.57 |
|  |  |  |  | Thr 480 | 3.62 |
|  |  |  |  | Gln 144 | 6.87 |
| 1GSK | Endosulfan diol | -5.9 | Van der Waals | Trp 463 |  |
|  |  |  |  | Pro 483 |  |
|  |  |  |  | Thr 480 |  |
|  |  |  |  | Arg 146 |  |
|  |  |  |  | Gln 144 |  |
|  |  |  |  | Gol 1512 |  |
|  |  |  |  | Arg 64 |  |
|  |  |  |  | Gln 145 |  |
|  |  |  |  | Val 110 |  |
|  |  |  |  | Gly 482 |  |
|  |  |  |  | Val 426 |  |
|  |  |  | Conventional Hydrogen Bond | Gln 362 | 5.46 |
|  |  |  |  | Ser 427 | 2.91 |
|  |  |  | Unfavorable aceptor-aceptor | Ser 427 | 3.18 |
|  | Endosulfan ether | -6 | Van der Waals | Ser 427 |  |
|  |  |  |  | Arg 146 |  |
|  |  |  |  | Gln 145 |  |
|  |  |  |  | Trp 463 |  |
|  |  |  |  | Val 110 |  |
|  |  |  |  | Pro 483 |  |
|  |  |  |  | Gly 482 |  |
|  |  |  |  | Thr 480 |  |
|  |  |  |  | Val 426 |  |
|  |  |  | Carbon Hydrogen Bond | Gln 144 | 6.44 |
|  | Endosulfan Hydroxyether | -6 | Van der Waals | Ser 427 |  |
|  |  |  |  | Arg 146 |  |
|  |  |  |  | Gln 145 |  |
|  |  |  |  | Trp 463 |  |
|  |  |  |  | Val 110 |  |
|  |  |  |  | Pro 483 |  |
|  |  |  |  | Gly 482 |  |
|  |  |  |  | Thr 480 |  |
|  |  |  |  | Val 426 |  |
|  |  |  |  | Gln 144 | 6.41 |
|  | Endosulfan Lactone | -6.6 | Van der Waals | Ser 427 |  |
|  |  |  |  | Arg 146 |  |
|  |  |  |  | Gln 145 |  |
|  |  |  |  | Trp 463 |  |
|  |  |  |  | Val 110 |  |
|  |  |  |  | Pro 483 |  |
|  |  |  |  | Gly 482 |  |
|  |  |  |  | Thr 480 |  |
|  |  |  |  | Val 426 |  |
|  |  |  | Carbon Hydrogen Bond | Gln 144 | 6.44 |
| 1HDH | Endosulfan | -6.6 | Van der Waals | Glu 326 |  |
|  |  |  |  | Trp 358 |  |
|  |  |  |  | Tyr 359 |  |
|  |  |  |  | Gly 360 |  |
|  |  |  |  | Pro 361 |  |
|  |  |  |  | Thr 262 |  |
|  |  |  |  | Arg 362 |  |
|  |  |  |  | Pro 232 |  |
|  |  |  |  | Arg 263 |  |
|  |  |  |  | Leu 268 |  |
|  |  |  |  | Glu 272 |  |
|  |  |  |  | Leu 324 |  |
|  |  |  | Conventional Hydrogen Bond | Ser 276 | 4.4 |
|  |  |  |  | Trp 363 | 4.82 |
|  |  |  | Carbon Hydrogen Bond | Arg 263 | 5.85 |
|  |  |  | Pi-Donor Hydrogen Bond | Ser 276 | 3.16 |
|  |  |  | Pi-Sulfur | Trp 265 | 5.94 |
|  | Endosulfan Diol | -5.9 | Van der Waals | Leu 336 |  |
|  |  |  |  | Asn 345 |  |
|  |  |  |  | Val 357 |  |
|  |  |  |  | Ile 360 |  |
|  |  |  |  | Arg 278 |  |
|  |  |  |  | Ala 279 |  |
|  |  |  |  | Ser 276 |  |
|  |  |  |  | Trp 363 |  |
|  |  |  |  | Trp 368 |  |
|  |  |  |  | Leu 324 |  |
|  |  |  |  | Glu 326 |  |
|  |  |  | Conventional Hydrgogen Bond | Lys 275 | 4.53 |
|  |  |  |  | Tyr 359 | 5.6 |
|  | Endosulfan Ether | -6 | Van der Waals | Leu 336 |  |
|  |  |  |  | Glu 326 |  |
|  |  |  |  | Asn 345 |  |
|  |  |  |  | Ile 360 |  |
|  |  |  |  | Ser 276 |  |
|  |  |  |  | Ala 279 |  |
|  |  |  |  | Val 357 |  |
|  |  |  |  | Trp 358 |  |
|  |  |  |  | Leu 324 |  |
|  | Endosulfan hydroxyether | -6 | Van der Waals | Leu 336 |  |
|  |  |  |  | Glu 326 |  |
|  |  |  |  | Asn 345 |  |
|  |  |  |  | Lys 275 |  |
|  |  |  |  | Ile 360 |  |
|  |  |  |  | Ser 276 |  |
|  |  |  |  | Ala 279 |  |
|  |  |  |  | Val 357 |  |
|  |  |  |  | Trp 358 |  |
|  |  |  |  | Leu 324 |  |
|  | Endosulfan Lactone | -6.6 | Van der Waals | Arg 421 |  |
|  |  |  |  | Asp 416 |  |
|  |  |  |  | Gly 437 |  |
|  |  |  |  | Pro 412 |  |
|  |  |  |  | Glu 434 |  |
|  |  |  |  | Leu 415 |  |
|  |  |  |  | His 422 |  |
|  |  |  | Conventional Hydrgogen Bond | Ser 439 | 4.11 |
|  |  |  |  | Arg 438 | 3.64 |
|  |  |  | Carbon Hydrogen Bond | Ser 439 | 4.38 |
|  |  |  |  | Pro 435 | 4.34 |
| 3BQ5 | Endosulfan | -7 | Van der Waals | Lys 104 |  |
|  |  |  |  | Trp 592 |  |
|  |  |  |  | Ile 587 |  |
|  |  |  |  | Pro 586 |  |
|  |  |  |  | Asn 107 |  |
|  |  |  |  | Cys 205 |  |
|  |  |  |  | Leu 156 |  |
|  |  |  | Conventional Hydrogen Bond | Arg 165 | 3.12 |
|  |  |  |  | Arg 165 | 2.99 |
|  |  |  | Carbon Hydrogen Bond | Tyr 155 | 3.72 |
|  |  |  | Unfavorable Positive-positive | Arg 165 | 3.79 |
|  |  |  | Pi-Cation | Phe 106 | 4.52 |
|  |  |  | Pi-sulfur | Tyr 155 | 4.91 |
|  | Endosulfan diol | -5.5 | Van der Waals | Thr 108 |  |
|  |  |  |  | Asn 107 |  |
|  |  |  |  | Trp 592 |  |
|  |  |  |  | Ala 585 |  |
|  |  |  |  | Arg 582 |  |
|  |  |  |  | Pro 586 |  |
|  |  |  |  | Phe 105 |  |
|  |  |  | Conventional Hydrogen Bond | Phe 106 | 2.21 |
|  |  |  |  | Phe 106 | 1.99 |
|  |  |  | Carbon Hydrogen Bond | Ile 587 | 3.42 |
|  | Endosulfan ether | -5.8 | Van der Waals | Arg 645 |  |
|  |  |  |  | Ser 673 |  |
|  |  |  |  | Pro 677 |  |
|  |  |  |  | Trp 669 |  |
|  |  |  |  | Val 668 |  |
|  |  |  |  | Ile 685 |  |
|  |  |  | Carbon Hydrogen Bond | Lys 647 | 3.47 |
|  |  |  |  | Ser 644 | 3.43 |
|  |  |  |  | Glu 681 | 3.26 |
|  | Endosulfan hydroxyehter | -5.8 | Van der Waals | Arg 645 |  |
|  |  |  |  | Ser 673 |  |
|  |  |  |  | Asp 670 |  |
|  |  |  |  | Ala 675 |  |
|  |  |  |  | Trp 669 |  |
|  |  |  |  | Val 668 |  |
|  |  |  |  | Ile 685 |  |
|  |  |  |  | Pro 677 |  |
|  |  |  | Carbon Hydrogen Bond | Glu 681 | 3.58 |
|  |  |  |  | Lys 647 | 3.48 |
|  |  |  |  | Ser 664 | 3.44 |
|  | Endosulfan Lactone | -6.2 | Van der Waals | Cys 205 |  |
|  |  |  |  | Trp 592 |  |
|  |  |  |  | Pro 586 |  |
|  |  |  |  | Leu 156 |  |
|  |  |  |  | Trp 163 |  |
|  |  |  | Conventional Hydrogen Bond | Arg 165 | 3.19 |
|  |  |  |  | Arg 165 | 3.12 |
| 3C7E | ENDOSULFAN | -7.6 | Van der Waals | Leu 28 |  |
|  |  |  |  | Ala 27 |  |
|  |  |  |  | Ala 292 |  |
|  |  |  |  | Leu229 |  |
|  |  |  |  | Phe 294 |  |
|  |  |  |  | Trp 300 |  |
|  |  |  |  | Val 167 |  |
|  |  |  |  | Phe 167 |  |
|  |  |  |  | Ala 106 |  |
|  |  |  |  | Ile 105 |  |
|  |  |  |  | Val 166 |  |
|  |  |  |  | Thr 29 |  |
|  |  |  | Conventional Hydrogen Bond | Asn 295 | 4.84 |
|  |  |  |  | Asn 295 | 3.88 |
|  |  |  | Carbon Hydrogen Bond | Val 293 | 5.92 |
|  | Endosulfan diol | -6.6 | Van der Waals | Val 166 |  |
|  |  |  |  | Ile 105 |  |
|  |  |  |  | Leu 229 |  |
|  |  |  |  | Ala 106 |  |
|  |  |  |  | Phe 167 |  |
|  |  |  |  | Lys 231 |  |
|  |  |  |  | His 230 |  |
|  |  |  |  | Trp 300 |  |
|  |  |  |  | Val 293 |  |
|  |  |  |  | Ala 27 |  |
|  |  |  |  | Thr 29 |  |
|  |  |  |  | Leu 28 |  |
|  |  |  | Conventional Hydrogen Bond | Asn 295 | 4.81 |
|  |  |  |  | Asn 295 | 4.43 |
|  |  |  | Halogen (Cl, Br, I) | Val 168 | 4.28 |
|  | Endosulfan ether | -6.7 | Van der Waals | Leu 28 |  |
|  |  |  |  | Ile 105 |  |
|  |  |  |  | Phe 294 |  |
|  |  |  |  | Asn 295 |  |
|  |  |  |  | Trp 300 |  |
|  |  |  |  | Val 293 |  |
|  |  |  |  | Lys 231 |  |
|  |  |  |  | His 230 |  |
|  |  |  |  | Leu 229 |  |
|  |  |  |  | Phe 167 |  |
|  |  |  |  | Val 166 |  |
|  |  |  |  | Ala 292 |  |
|  |  |  |  | Val 168 |  |
|  |  |  |  | Ala 27 |  |
|  |  |  |  | Thr 29 |  |
|  | Endosulfan Hydroxyether | -6.7 | Van der Waals | Leu 28 |  |
|  |  |  |  | Ile 105 |  |
|  |  |  |  | Phe 294 |  |
|  |  |  |  | Asn 295 |  |
|  |  |  |  | Trp 300 |  |
|  |  |  |  | Val 293 |  |
|  |  |  |  | Lys 231 |  |
|  |  |  |  | His 230 |  |
|  |  |  |  | Leu 229 |  |
|  |  |  |  | Phe 167 |  |
|  |  |  |  | Val 166 |  |
|  |  |  |  | Ala 292 |  |
|  |  |  |  | Val 168 |  |
|  |  |  |  | Ala 27 |  |
|  |  |  |  | Thr 29 |  |
|  | Endosulfan Lactone | -7 | Van der Waals | Ala 27 |  |
|  |  |  |  | Thr 29 |  |
|  |  |  |  | Ile 105 |  |
|  |  |  |  | Ala 106 |  |
|  |  |  |  | Trp 300 |  |
|  |  |  |  | Lys 231 |  |
|  |  |  |  | Phe 167 |  |
|  |  |  |  | Val 293 |  |
|  |  |  |  | Val 166 |  |
|  |  |  |  | Val 168 |  |
|  |  |  |  | Ala 292 |  |
|  |  |  |  | Leu 28 |  |
|  |  |  | Conventional Hydrogen Bond | Asn 295 | 4.36 |
|  |  |  | Carbon Hydrogen Bond | Phe 294 |  |
|  |  |  |  | Leu 229 | 6.37 |
| 3D30 | Endosulfan | -5.9 | Van der Waals | Thr 115 |  |
|  |  |  |  | Arg 132 |  |
|  |  |  |  | Ile 110 |  |
|  |  |  |  | Tyr 55 |  |
|  |  |  |  | Met 32 |  |
|  |  |  | Conventional Hydrogen Bond | Asn 133 | 3.65 |
|  |  |  | Carbon Hydrogen Bond | Pro 27 | 4.11 |
|  |  |  |  | Tyr 68 | 7.3 |
|  |  |  |  | Tyr 68 | 5.75 |
|  |  |  |  | Pro 29 | 4.7 |
|  |  |  | Pi-Cation | Tyr 68 | 5.85 |
|  | Endosulfan diol | -5.2 | Van der Waals | Pro 29 |  |
|  |  |  |  | Tyr 68 |  |
|  |  |  |  | Ile 28 |  |
|  |  |  |  | Thr 115 |  |
|  |  |  | Conventional Hydrogen Bond | Asn 133 | 3.95 |
|  |  |  |  | Leu 25 | 3.49 |
|  |  |  | Carbon Hydrogen Bond | Pro 27 | 3.95 |
|  |  |  |  | Pro 29 | 4.86 |
|  | Endosulfan ether | -5.4 | Van der Waals | Pro 27 |  |
|  |  |  |  | Ile 28 |  |
|  |  |  |  | Tyr 68 |  |
|  |  |  |  | Met 32 |  |
|  |  |  |  | Ile 110 |  |
|  |  |  |  | Tyr 55 |  |
|  |  |  |  | Leu 25 |  |
|  |  |  | Conventional Hydrogen Bond | Asn 133 | 3.74 |
|  |  |  |  | Arg 132 | 3.74 |
|  | Endosulfan Hydroxyether | -5.9 | Van der Waals | Pro 27 |  |
|  |  |  |  | Ile 28 |  |
|  |  |  |  | Tyr 68 |  |
|  |  |  |  | Met 32 |  |
|  |  |  |  | Ile 110 |  |
|  |  |  |  | Tyr 55 |  |
|  |  |  |  | Leu 25 |  |
|  |  |  | Conventional Hydrogen Bond | Asn 133 | 3.73 |
|  |  |  | Carbon Hydrogen Bond | Pro 29 | 5.05 |
|  |  |  |  | Arg 132 | 3.72 |
|  | Endosulfan Lactone | -5.8 | Van der Waals | Ile 28 |  |
|  |  |  |  | Thr 115 |  |
|  |  |  |  | Tyr 68 |  |
|  |  |  |  | Met 32 |  |
|  |  |  |  | Pro 27 |  |
|  |  |  | Conventional Hydrogen Bond | Asn 133 | 3.62 |
|  |  |  |  | Tyr 55 | 6.9 |
|  |  |  | Carbon Hydrogen Bond | Arg 132 | 3.74 |
|  |  |  |  | Arg 132 | 3.84 |
|  |  |  |  | Pro 29 | 4.84 |
|  |  |  | Halogen (Cl Br, I) | Leu 25 | 4.38 |
| 3L65 | Endosulfan | -6.9 | Van der Waals | Phe 269 |  |
|  |  |  |  | Lys 106 |  |
|  |  |  |  | His 181 |  |
|  |  |  |  | Tyr 183 |  |
|  |  |  |  | Leu 141 |  |
|  |  |  |  | Ile 66 |  |
|  |  |  |  | Tyr 27 |  |
|  |  |  |  | Pro 12 |  |
|  |  |  |  | His 140 |  |
|  |  |  | Attractive charge | Asp 116 | 5.66 |
|  | Endosulfan diol | -5.9 | Van der Waals | Glu 345 |  |
|  |  |  |  | Val 344 |  |
|  |  |  |  | Thr 350 |  |
|  |  |  |  | Phe 5 |  |
|  |  |  |  | Asn 17 |  |
|  |  |  |  | Ser 348 |  |
|  |  |  | Conventional Hydrogen Bond | Lys 346 | 5.76 |
|  |  |  | Carbon Hydrogen Bond | Ala 347 | 4.29 |
|  |  |  | Halogen (Cl, Br, I) | Arg 16 | 5.5 |
|  |  |  | Unfavorable Aceptor-Aceptor | Trp 349 | 6.01 |
|  | Endosulfan ether | -6 | Van der Waals | Phe 269 |  |
|  |  |  |  | Ser 266 |  |
|  |  |  |  | Trp 278 |  |
|  |  |  |  | Trp 302 |  |
|  |  |  |  | Met 24 |  |
|  |  |  |  | Pro 22 |  |
|  |  |  |  | Arg 231 |  |
|  |  |  |  | Pro 23 |  |
|  |  |  |  | Ala 301 |  |
|  |  |  | Van der Waals | Asn 17 |  |
|  |  |  |  | Arg 16 |  |
|  |  |  |  | Trp 349 |  |
|  |  |  | Carbon Hydrogen Bond | Lys 346 | 6.44 |
|  | Endosulfan Hydroxyether | -6 | Van der Waals | Pro 23 |  |
|  |  |  |  | Ala 301 |  |
|  |  |  |  | His 181 |  |
|  |  |  |  | Phe 269 |  |
|  |  |  |  | Ser 266 |  |
|  |  |  |  | Trp 278 |  |
|  |  |  |  | Trp 302 |  |
|  |  |  |  | Met 24 |  |
|  |  |  |  | Pro 22 |  |
|  |  |  |  | Arg 231 |  |
|  |  |  |  | Pro 23 |  |
|  | Endosulfan Lactone | -6.3 | Van der Waals | Phe 137 |  |
|  |  |  |  | Asp 116 |  |
|  |  |  |  | Leu 141 |  |
|  |  |  |  | His 181 |  |
|  |  |  |  | Phe 269 |  |
|  |  |  |  | Lys 106 |  |
|  |  |  | Conventional Hydrogen Bond | Tyr 183 | 5.35 |
|  |  |  | Carbon Hydrogen Bond | His 140 | 5.65 |
| 3N2S | Endosulfan | -4.9 | Van der Waals | Asn 140 |  |
|  |  |  |  | Tyr 236 |  |
|  |  |  |  | Arg 234 |  |
|  |  |  |  | Lys 232 |  |
|  |  |  |  | Pro 233 |  |
|  |  |  |  | Glu 141 |  |
|  |  |  | Conventional Hydrogen Bond | Thr 235 | 3.33 |
|  | Endosulfan Diol | -3.9 | Van der Waals | Glu 141 |  |
|  |  |  |  | Lys 232 |  |
|  |  |  |  | Pro 233 |  |
|  |  |  |  | Arg 234 |  |
|  |  |  | Conventional Hydrogen Bond | Thr 235 | 3.29 |
|  |  |  |  | Tyr 236 | 4.31 |
|  |  |  | Unfavorable Aceptor-Aceptor | Asn 140 | 6.2 |
|  | Endosulfan Ether | -4 | Van der Waals | Tyr 240 |  |
|  |  |  |  | Asp 143 |  |
|  |  |  |  | Tyr 236 |  |
|  |  |  | Carbon Hydrogen Bond | Glu 141 | 3.94 |
|  |  |  |  | Asn 140 | 6.49 |
|  | Endosulfan Hydroxyether | -3.9 | Van der Waals | Phe 228 |  |
|  |  |  |  | Arg 234 |  |
|  | Endosulfan Lactone | -4.4 | Van der Waals | Glu 141 |  |
|  |  |  |  | Tyr 236 |  |
|  |  |  |  | Tyr 240 |  |
|  |  |  |  | Asp 148 |  |
|  |  |  | Conventional Hydrogen Bond | Asn 140 | 6.23 |
|  |  |  | Carbon Hydrogen Bond | Lys 144 | 5.15 |
| 3RP8 | Endosulfan | -5.7 | Van der Waals | Arg 277 |  |
|  |  |  |  | Gly 166 |  |
|  |  |  |  | Pro 162 |  |
|  |  |  |  | Arg 127 |  |
|  |  |  |  | Trp 163 |  |
|  |  |  | Carbon Hydrogen Bond | Gln 314 | 4.22 |
|  | Endosulfan diol | -5.1 | Van der Waals | Thr | 366 |
|  |  |  |  | Glu 368 |  |
|  |  |  |  | Gly 367 |  |
|  |  |  |  | Glu 365 |  |
|  |  |  |  | Glu 361 |  |
|  |  |  |  | Glu 364 |  |
|  |  |  | Carbon Hydrogen Bond | Arg 369 | 4.33 |
|  | Endosulfan Ether | -4.6 | Van der Waals | Arg 97 |  |
|  |  |  |  | Gly 95 |  |
|  |  |  |  | Gly 58 |  |
|  |  |  |  | Glu 62 |  |
|  |  |  |  | Met 61 |  |
|  |  |  |  | Val 49 |  |
|  |  |  |  | Ser 96 |  |
|  |  |  |  | Ala 53 |  |
|  |  |  |  | Lys 50 |  |
|  | Endosulfan hydroxyether | -4.6 | Van der Waals | Pro 89 |  |
|  |  |  |  | Glu 368 |  |
|  |  |  |  | Ile 371 |  |
|  |  |  |  | Asn 372 |  |
|  |  |  | Conventional Hydrogen Bond | Arg 93 | 4.14 |
|  |  |  |  | Arg 93 | 5.23 |
|  | Endosulfan Lactone | -5 | Van der Waals | Ala 53 |  |
|  |  |  |  | Lys 50 |  |
|  |  |  |  | Glu 62 |  |
|  |  |  |  | Met 61 |  |
|  |  |  |  | Gly 58 |  |
|  |  |  |  | Val 49 |  |
| 3ZDW | Endosulfan | -6.6 | Van der Waals | Phe 228 |  |
|  |  |  |  | Val 225 |  |
|  |  |  |  | Ala 211 |  |
|  |  |  |  | Pro 212 |  |
|  |  |  |  | Pro 209 |  |
|  |  |  |  | Ala 227 |  |
|  |  |  | Attractive Charge | Glu 213 | 6.71 |
|  |  |  | Conventional Hydrogen Bond | Cys 229 | 4.35 |
|  |  |  | Carbon Hydrogen Bond | Pro 226 | 4.48 |
|  | Endosulfan Diol | -5.7 | Van der Waals | Pro 384 |  |
|  |  |  |  | Leu 386 |  |
|  |  |  |  | Ala375 |  |
|  |  |  |  | Gln 442 |  |
|  |  |  |  | Arg 416 |  |
|  |  |  | Conventional Hydrogen Bond | Val 385 | 4.86 |
|  |  |  |  | Thr 377 | 4.73 |
|  |  |  |  | Gly 376 | 3.2 |
|  |  |  | Carbon Hydrogen Bond | Thr 415 | 4.03 |
|  | Endosulfan Ether | -5.5 | Van der Waals | Glu 213 |  |
|  |  |  |  | Ala 211 |  |
|  |  |  |  | Pro 212 |  |
|  |  |  |  | Pro 209 |  |
|  |  |  |  | Val 225 |  |
|  |  |  |  | Phe 228 |  |
|  |  |  |  | Ala 227 |  |
|  |  |  | Carbon Hydrogen Bond | Pro 226 | 4.4 |
|  |  |  | Halogen (Cl, Br, I) | Cys 229 | 3.96 |
|  | Endosulfan Hydroxyether | -5.5 | Van der Waals | Cys 229 |  |
|  |  |  |  | Ala 227 |  |
|  |  |  |  | Glu 213 |  |
|  |  |  |  | Ala 211 |  |
|  |  |  |  | Pro 212 |  |
|  |  |  |  | Pro 209 |  |
|  |  |  |  | Phe 228 |  |
|  |  |  |  | Val 225 |  |
|  |  |  | Carbon Hydrogen Bond | Pro 226 | 4.45 |
|  | Endosulfan Lactone | -6 | Van der Waals | Pro 209 |  |
|  |  |  |  | Pro 212 |  |
|  |  |  |  | Glu 213 |  |
|  |  |  |  | Gly 323 |  |
|  |  |  |  | Ala 227 |  |
|  |  |  |  | Val 225 |  |
|  |  |  | Conventional Hydrogen Bond | Cys 229 | 3.45 |
|  |  |  |  | Pro 226 | 3.98 |
| 4eti | Endosulfan | -5 | Van der Waals | Met 1 |  |
|  |  |  |  | Leu 79 |  |
|  |  |  |  | Lys 101 |  |
|  |  |  |  | Asp 100 |  |
|  |  |  |  | Phe 103 |  |
|  |  |  |  | Arg 150 |  |
|  |  |  |  | Asp 78 |  |
|  |  |  | Attractive charge | Asp 148 | 7.24 |
|  |  |  | Conventional Hydrogen Bond | Tyr 108 | 6.44 |
|  |  |  | Pi-Sulfur | Tyr 108 | 6.53 |
|  | Endosulfan Diol | -5 | Van der Waals | Lys 22 |  |
|  |  |  |  | Ser 36 |  |
|  |  |  |  | Asn 10 |  |
|  |  |  |  | His 64 |  |
|  |  |  |  | Ser 67 |  |
|  |  |  |  | Leu 73 |  |
|  |  |  | Conventional Hydrogen Bond | Glu 18 | 5.27 |
|  |  |  |  | Glu 18 | 4.77 |
|  |  |  |  | Val 34 | 5.32 |
|  |  |  | Carbon Hydrogen Bond | Arg 35 | 4.99 |
|  |  |  | Halogen (Cl, Br, I) | Val 65 | 4.71 |
|  | Endosulfan ether | -4.9 | Van der Waals | Lys 106 |  |
|  |  |  |  | Leu 117 |  |
|  |  |  |  | Thr 130 |  |
|  |  |  |  | Val 116 |  |
|  |  |  |  | Glu 133 |  |
|  |  |  |  | Gln 129 |  |
|  |  |  |  | Ile 126 |  |
|  | Endosulfan Hydroxyether | -4.9 | Van der Waals | Gln 129 |  |
|  |  |  |  | Thr 130 |  |
|  |  |  |  | Leu 117 |  |
|  |  |  |  | Val 116 |  |
|  |  |  | Carbon Hydrogen Bond | Glu 133 | 5.99 |
|  |  |  | Halogen (Cl, Br, I) | Ile 126 | 3.94 |
|  | Endosulfan Lactone | -5.2 | Van der Waals |  |  |
|  |  |  |  | Leu 117 |  |
|  |  |  |  | Gln 86 |  |
|  |  |  |  | Thr 84 |  |
|  |  |  |  | Thr 84 |  |
|  |  |  |  | Arg 13 |  |
|  |  |  |  | Asp 118 |  |
|  |  |  |  | Leu 117 |  |
|  |  |  |  | His 87 | 4.09 |
| 7wyg | Endosulfan | -6.6 | Van der Waals | Phe 289 |  |
|  |  |  |  | Gln 352 |  |
|  |  |  |  | Cys 363 |  |
|  |  |  |  | His 361 |  |
|  |  |  |  | Phe 79 |  |
|  |  |  |  | Ile 78 |  |
|  |  |  |  | Ala 246 |  |
|  |  |  |  | Arg 242 |  |
|  |  |  |  | Pro 243 |  |
|  |  |  | Unfavorable Positive-Positive | His 85 | 6.28 |
|  | Endosulfan Diol | -5.1 | Van der Waals | Phe 289 |  |
|  |  |  |  | Ile 290 |  |
|  |  |  |  | Gln 352 |  |
|  |  |  |  | Cys 363 |  |
|  |  |  |  | Pro 364 |  |
|  |  |  |  | Gly 365 |  |
|  |  |  |  | His 85 |  |
|  |  |  |  | Arg 242 |  |
|  |  |  |  | Val 170 |  |
|  |  |  |  | Pro 243 |  |
|  |  |  |  | Phe 79 |  |
|  |  |  |  | Ala 246 |  |
|  |  |  |  | Ile 78 |  |
|  |  |  |  | Leu 293 |  |
|  | Endosulfan ether | -6.6 | Van der Waals | Ala 246 |  |
|  |  |  |  | Ile 78 |  |
|  |  |  |  | Phe 289 |  |
|  |  |  |  | Val 170 |  |
|  |  |  |  | Pro 243 |  |
|  |  |  |  | Arg 242 |  |
|  |  |  |  | Arg 242 |  |
|  |  |  |  | His 85 |  |
|  |  |  |  | Cys 363 |  |
|  |  |  |  | Gln 352 |  |
|  |  |  |  | Leu 293 |  |
|  |  |  |  | Ile 290 |  |
|  | Endosulfan Hydroxyether | -6.6 | Van der Waals | Phe 289 |  |
|  |  |  |  | Val 170 |  |
|  |  |  |  | Phe 79 |  |
|  |  |  |  | Val 170 |  |
|  |  |  |  | Pro 243 |  |
|  |  |  |  | Arg 242 |  |
|  |  |  |  | His 85 |  |
|  |  |  |  | Cys 363 |  |
|  |  |  |  | Gln 352 |  |
|  |  |  |  | Leu 293 |  |
|  |  |  |  | Ile 290 |  |
|  |  |  |  | Ala 246 |  |
|  |  |  |  | Ile 78 |  |
|  | Endosulfan Lactone | -5.7 | Van der Waals | Ser 273 |  |
|  |  |  |  | Lys 109 |  |
|  |  |  |  | Lys 109 |  |
|  |  |  |  | Glu 371 |  |
|  |  |  |  | Lys 374 |  |
|  |  |  |  | Ala 375 |  |
|  |  |  |  | Asp 378 |  |
|  |  |  |  | Thr 116 |  |
|  |  |  |  | Glu 113 |  |
|  |  |  | Halogen (Cl, Br, I) | Ala 112 | 3.79 |
| 8HKD | ENDOSULFAN | -5.6 | Van der Waals | Lys 109 |  |
|  |  |  |  | Ala 112 |  |
|  |  |  |  | Glu 113 |  |
|  |  |  |  | Thr 116 |  |
|  |  |  |  | Ala 375 |  |
|  |  |  |  | Arg 276 |  |
|  |  |  | Conventional Hydrogen Bond | Lys 374 | 5.02 |
|  |  |  |  | Glu 371 | 5.72 |
|  |  |  | Attractive charge | Glu 371 | 6.67 |
|  | Endosulfan Diol | -5 | Van der Waals | Glu 341 |  |
|  |  |  |  | Glu 344 |  |
|  |  |  |  | Phe 339 |  |
|  |  |  |  | Gln 281 |  |
|  |  |  |  | Met 278 |  |
|  |  |  |  | Glu 277 |  |
|  |  |  |  | Ala 340 |  |
|  |  |  |  | Arg 274 |  |
|  |  |  | Conventional Hydrogen Bond | Arg 342 |  |
|  |  |  |  | Arg 342 | 4.63 |
|  |  |  |  |  | 5.19 |
|  | Endosulfan ether | -5 | Van der Waals | Asp 387 |  |
|  |  |  |  | Val 388 |  |
|  |  |  |  | Pro 262 |  |
|  |  |  |  | Leu 258 |  |
|  |  |  |  | Tyr 386 |  |
|  |  |  | Carbon Hydrogen Bond | Lys 265 | 3.59 |
|  |  |  | Conventional Hydrogen Bond | Lys 265 | 2 |
|  |  |  | Halogen (Cl, Br, I) | His 259 | 3.22 |
|  | Endosulfan Hydroxyether | -5.3 | Van der Waals | Lys 109 |  |
|  |  |  |  | Glu 113 |  |
|  |  |  |  | Ala 112 |  |
|  |  |  |  | Thr 116 |  |
|  |  |  |  | Lys 374 |  |
|  |  |  |  | Ala 375 |  |
|  |  |  |  | Asp 378 |  |
|  |  |  | Conventional Hydrogen Bond | Arg 276 | 2.7 |
|  |  |  | Carbon Hydrogen Bond | Glu 371 | 3.48 |
|  | Endosulfan Lactone | -5.8 | Van der Waals | Thr 116 |  |
|  |  |  |  | Asp 378 |  |
|  |  |  |  | Ala 375 |  |
|  |  |  |  | Arg 276 |  |
|  |  |  |  | Lys 374 |  |
|  |  |  |  | Ala 112 |  |
|  |  |  |  | Lys 109 |  |
|  |  |  |  | Glu 113 |  |
|  |  |  | Carbon Hydrogen Bond | Glu 371 | 3.38 |
|  |  |  |  |  |  |
|  |  |  |  |  |  |
